# Associations between accelerometry measured physical activity and sedentary time and the metabolic syndrome: A meta‐analysis of more than 6000 children and adolescents

**DOI:** 10.1111/ijpo.12578

**Published:** 2019-11-10

**Authors:** Marius Renninger, Bjørge H. Hansen, Jostein Steene‐Johannessen, Susi Kriemler, Karsten Froberg, Kate Northstone, Luis Sardinha, Sigmund A. Anderssen, Lars B. Andersen, Ulf Ekelund, On behalf of the International Children's Accelerometry Database (ICAD) Collaborators

**Affiliations:** ^1^ Department of Sport Medicine Norwegian School of Sport Sciences Oslo Norway; ^2^ Department of Medical Informatics, Biometry and Epidemiology (IBE) Ludwig‐Maximilians‐Universität München Munich Germany; ^3^ Epidemiology, Biostatistics and Public Health Institute University of Zürich Zurich Switzerland; ^4^ Center of Research in Childhood Health (RICH) University of Southern Denmark Odense Denmark; ^5^ School of Social and Community Medicine University of Bristol Bristol UK; ^6^ Exercise and Health Laboratory, CIPER, Fac Motricidade Humana Universidade de Lisboa Lisbon Portugal; ^7^ Faculty of Education, Arts and Sport Western Norway University of Applied Sciences Sogndal Norway; ^8^ Norwegian Institute for Public Health Oslo Norway

**Keywords:** Metabolic syndrome, obesity, physical activity, sedentary behaviour

## Abstract

**Background:**

Metabolic syndrome is increasingly prevalent in the pediatric population. To prevent an early onset, knowledge about its association with modifiable lifestyle factors is needed.

**Objectives:**

To estimate the prevalence of the metabolic syndrome and examine its cross‐sectional associations with physical activity and sedentary time.

**Methods:**

Participants were 6009 children and adolescents from 8 studies of the International Children's Accelerometry Database. Physical activity and sedentary time were measured by accelerometer. Metabolic syndrome was defined based on International Diabetes Federation criteria. Logistic regression models adjusted for sex, age and monitor wear time were used to examine the associations between physical activity, sedentary time and the metabolic syndrome in each study and effect estimates were combined using random‐effects meta‐analysis.

**Results:**

The overall prevalence of the metabolic syndrome was 2.9%. In crude models, a 10 min increase in moderate‐to‐vigorous intensity physical activity and vigorous‐intensity physical activity were inversely associated with the metabolic syndrome [OR 0.88, 95% CI 0.82‐0.94, OR 0.80, 95% CI 0.70‐0.92]. One hour increase in sedentary time was positively associated with the metabolic syndrome [OR 1.28, 95% CI 1.13‐1.45]. After adjustment for sedentary time, the association between moderate‐to‐vigorous‐intensity physical activity and the metabolic syndrome remained significant [OR 0.91, 95% CI 0.84‐0.99]. Sedentary time was not associated with the metabolic syndrome after adjustment for moderate‐to‐vigorous intensity physical activity [OR 1.14 95% CI 0.96‐1.36].

**Conclusions:**

Physical activity of at least moderate intensity but not sedentary time is independently associated with the metabolic syndrome.

## 1 INTRODUCTION

Although the prevalence of the metabolic syndrome (MetS) in children and adolescents is low, it rises with age.^1^, ^2^ Furthermore MetS is more prevalent in youth with overweight and obesity^3^ which is especially threatening with regard to the continuous rise of obesity levels in this population.^4^ Cardiovascular disease (CVD) risk factors already begin to cluster during childhood and adolescence^5^ and track into adulthood.^6^ Evidence from longitudinal studies suggests that pediatric MetS may transfer into an increased risk for CVD, type 2 diabetes mellitus and premature death.^7^, ^8^, ^9^ To develop effective public health strategies and prevent an early onset of MetS, knowledge about preventive lifestyle factors such as physical activity and time spent sedentary is required.

Existing evidence in children and adolescents indicates a beneficial association between objectively measured physical activity and several cardio‐metabolic biomarkers as well as adiposity.^10^ The association between sedentary time and cardio‐metabolic health remains inconsistent. A recent systematic review suggested strong evidence for a prospective relationship between TV‐viewing time and obesity and for an inverse relationship between different sedentary time measures and HDL‐cholesterol but no or insufficient evidence for associations between sedentary time and other biomarkers and metabolic risk scores.^11^ When total sedentary time measured by accelerometry is considered, some found cross‐sectional associations between sedentary time and a metabolic risk score^12^, ^13^ or single risk factors^14^ while others did not.^15^, ^16^ Further, associations between sedentary time and metabolic risk scores appear attenuated when additionally adjusted for physical activity.^17^, ^18^, ^19^ Prospectively, no association between sedentary time and a metabolic risk score or single risk factors have been observed.^20^, ^21^

However, most studies with objective measurements of physical activity and sedentary time have used metabolic risk scores or single biomarkers as outcomes and only three studies have assessed the associations between objectively measured physical activity and MetS^1^, ^22^, ^23^ and only one of these examined the associations between objectively measured sedentary time and MetS as a dichotomous outcome in children and adolescents.^23^ The advantage of using MetS as a dichotomous variable rather than a continuous score is that MetS prevalence and effect estimates (e.g. OR, RR) can be compared across studies and countries.

The objectives of this study were therefore to estimate the prevalence of MetS and examine the associations between objectively measured physical activity, sedentary time and MetS in a large and diverse multicentre sample of 6009 children and adolescents using a meta‐analytical approach.

## 2 METHODS

### 2.1 Study design

The International Children's Accelerometry Database (ICAD) is a database of pooled data on objectively measured physical activity from 21 studies in children and adolescents worldwide. In 2008, 19 studies with a sample size > 400 which used any version of the ActiGraph accelerometer (ActiGraph LLC, Pensacola, FL) in children 3‐18 years were identified through a PubMed search and six additional studies through personal contacts. Finally, 21 studies contributed data to the ICAD. Detailed information on the methods of the ICAD project can be found elsewhere.^24^ All participants and/or their legal guardian provided written informed consent and local ethical committees approved the study protocols. Prior to sharing data, data‐sharing agreements were established between contributing studies and MRC Epidemiology Unit, University of Cambridge, UK.

### 2.2 Participants

For the present analysis we used data from 8 studies from Europe and the United States, which provided data on objectively measured physical activity and comparable fasting blood samples.^25^, ^26^, ^27^, ^28^, ^29^, ^30^ The included studies' designs are longitudinal,^28^, ^29^ cross‐sectional^25^, ^26^, ^28^ and interventional^27^, ^30^ respectively. Data was only used at one point in time for longitudinal and interventional studies. Thus, the present analyses are cross‐sectional in nature.

The dataset consisted of 15 068 boys and girls aged 9‐18 years. After excluding individuals with invalid accelerometer measurements (n=477), missing data on weight (n=56) and height (n=6) and incomplete information on features of MetS (n=8520) the final sample included 6009 individuals. In those individuals with multiple measurements on exposure and outcome variables we included the most recent data.

### 2.3 Assessment of physical activity and sedentary time

Physical activity and sedentary time were measured using hip‐worn, uniaxial ActiGraph accelerometers (models 7164, 71256 and GT1M), previously validated for energy expenditure assessed by the doubly labelled water method in free living children.^31^ All available raw accelerometer files were centrally processed, cleaned and reanalyzed using Kinesoft software (Kinesoft, version 3.3.20), to provide physical activity variables that could be directly compared. Before analysis, all files with shorter epoch lengths than 60 seconds were reintegrated to 60 second epochs for comparability. Non‐wear time was considered as 60 minutes of consecutive zeros, allowing for two minutes of non‐zero interruptions. The valid day criterion for the present study was 480 minutes of measured wear time between 7 AM and midnight. Individuals with at least one valid day were included. To estimate the time spent in light (101‐2295 cpm), moderate (2296‐4011 cpm) and vigorous intensity (> 4011 cpm), as well as sedentary time (0‐100 cpm), Evenson cut‐points were used.^32^ Total physical activity was expressed in counts per minute (CPM). For each day, total accelerometer counts were divided by monitor wear time in minutes and then averaged across all valid days.

### 2.4 Assessment of the MetS

MetS was defined according to the IDF pediatric definition in children and adolescents aged 9 to 15 years and according to the IDF worldwide adult definition in adolescents aged 16 to 18 years.^33^, ^34^

The IDF pediatric definition defines MetS as having abdominal obesity (waist circumference ≥ 90^th^ age and sex specific percentile or adult cut‐off if lower) and the presence of two or more of the following clinical features: elevated triglycerides (≥ 1.7 mmol/L), low HDL‐cholesterol (< 1.03 mmol/L), high blood pressure (systolic ≥ 130/diastolic ≥ 85 mm Hg) and increased fasting plasma glucose (≥ 5.6 mmol/L) or known type 2 diabetes mellitus.^33^ Reference values were taken from a population‐based sample of British children.^35^

The IDF worldwide adult definition defines MetS as having abdominal obesity (≥ 94cm for Caucasian men and ≥ 80cm for Caucasian women) and the presence of two or more of the following clinical features: elevated triglycerides (≥ 1.7 mmol/L or specific treatment for this lipid abnormality), low HDL‐cholesterol (< 1.03 mmol/L in males and < 1.29 mmol/L in females or specific treatment for this lipid abnormality), high blood pressure (systolic ≥ 130/diastolic ≥ 85 mm Hg or treatment of previously diagnosed hypertension) and increased fasting plasma glucose(≥ 5.6 mmol/L) or known type 2 diabetes mellitus.^34^

Blood pressure was measured using standard procedures, described in detail elsewhere^19^, ^25^, ^26^, ^27^

Height and weight were measured using standardized clinical procedures across studies. Body mass index (BMI) was calculated as weight in kilograms divided by the square of height in meters (kg/m^2^). Except for the NHANES (National Health and Nutrition Examination Survey), waist circumference (WC) was measured at the end of gentle expiration midway between the lower rib margin and the iliac crest using a metal tape.^27^, ^28^, ^29^, ^30^ In NHANES WC was measured just above the iliac crest at the midaxillary line using similar equipment.^36^

Triglycerides, HDL cholesterol Insulin and plasma glucose were measured in all studies.^25^, ^26^, ^27^, ^28^, ^29^, ^30^ All blood samples were taken according to standard clinical procedures as previously described.^25^, ^26^, ^27^, ^28^, ^29^, ^30^

### 2.5 Statistical analysis

Differences between excluded and included individuals and between sexes were calculated by T‐test, Mann‐Whitney‐u‐test and chi‐square tests where appropriate.

Logistic regression models were used to examine the cross‐sectional associations between total physical activity (CPM), MVPA, VPA and sedentary time with MetS for each study separately. The models were adjusted for sex, age and monitor wear time. When total physical activity was modelled as the main exposure, monitor wear time was left out of the model because it is included in the calculation of the variable. Additionally, MVPA and sedentary time and VPA and sedentary time were mutually adjusted for each other. The resulting odds ratios represent a 10 min increase in MVPA or VPA, a 60 min increase in sedentary time and a 100 cpm increase in total physical activity. Thereafter, random effects meta‐analysis was used to combine odds ratios across all studies.^37^ Heterogeneity was determined by I^2^ statistics. I^2^ represents the variability in effect estimates due to heterogeneity rather than sampling error. Analyses were performed using IBM SPSS Statistics for Windows, version 24 (IBM Corp., Armonk, N.Y., USA) and meta‐analyses were conducted in Stata version 12 (StataCorp., LLC, College Station, TX, USA) using the Metan package.

Sensitivity analyses were conducted including individuals with at least 2, 3 and 4 valid days of accelerometer data.

## 3 RESULTS

Cohort Characteristics are presented in Table 1 and differences between included and excluded individuals are presented in Table S1 in the Supporting Information. Excluded individuals were slightly younger (age 13.5 year (1.9) vs. 14.0 year (2.6), p <0.001) and more active (TPA 533 cpm (212) vs. 501 cpm (226), p < 0.001).

**Table 1:** Descriptive statistics of children and adolescents by study

Study	ALSPAC	CoSCIS	EYHS Denmark	EYHS Estonia	EYHS Portugal	KISS	NHANES 03/04	NHANES 05/06
Country	England	Denmark	Denmark	Estonia	Portugal	Switzerland	USA	USA
Study design	Long.	Inter.	Long.	Cross.	Long.	Inter.	Cross.	Cross.
Boys (n)	559	197	726	276	366	98	358	296
Girls (n)	695	165	895	331	346	118	289	294
Age	15.5 (0.3)	9.6 (0.3)	13.5 (2.8)	12.7 (2.9)	13.1 (3.2)	11.4 (0.6)	15.2 (1.9)	15.1 (1.9)
Height, cm	169.1 (8.4)	139.9 (6.0)	159.4 (16.3)	154.1 (17.4)	151.9 (16.3)	148.2 (7.5)	165.4 (10.1)	164.1 (9.8)
Weight, kg	60.9 (10.7)	33.8 (6.5)	51.5 (15.7)	45.8 (16.0)	47.6 (16.0)	40.1 (8.5)	65.1 (19.2)	63.3 (18.8)
BMI	21.3 (3.2)	17.2 (2.5)	19.8 (3.3)	18.6 (3.1)	20.0 (3.7)	18.1 (2.7)	23.6 (5.9)	23.3 (5.9)
SBP (mm HG)	123.1 (10.7)	104.3 (8.9)	107.3 (11.0)	106.3 (11.4)	100.8 (10.6)	105.0 (8.4)	109.2 (10.5)	109.8 (9.6)
DBP (mm HG)	66.4 (8.7)	61.8 (6.1)	61.0 (6.3)	61.4 (7.1)	57.1 (6.7)	63.1 (7.2)	59.8 (10.5)	59.8 (10.4)
HDL (mmol/l)	1.3 (0.3)	1.6 (0.3)	1.4 (0.4)	1.4 (0.3)	1.5 (0.3)	1.7 (0.4)	1.4 (0.3)	1.4 (0.3)
Glucose (mmol/l)	5.2 (0.4)	4.9 (0.5)	5.0 (0.4)	5.1 (0.4)	5.2 (0.4)	4.7 (0.4)	5.0 (0.5)	5.2 (0.6)
Insulin (pmol/l)	61.6 (45.9‐82.0)	37.5 (27.8‐50.0)	52.2 (37.2‐71.3)	49.6 (33.5‐71.3)	38.4 (27.1‐52.5)	58.3 (41.0‐74.3)	62.4 (41.5‐100.6)	69.5 (46.6‐103.3)
TG (mmol/l)	0.73 (0.59‐0.96)	0.50 (0.40‐0.7)	0.77(0.56‐1.05)	0.70 (0.55‐0.92)	0.62 (0.46‐0.85	0.60 (0.44‐0.77)	0.82 (0.60‐1.11)	0.80 (0.59‐1.07)
WC (cm)	75.2 (71.1‐81.0)	60.7 (57.5‐64.6)	68.0 (61.8‐73.4)	62.3 (56.6‐68.5)	66.0 (60.3‐72.0)	61.5 (58.0‐65.5)	77.2 (70.0‐87.9)	76.8 (70.0‐87.6)
MetS cases n (%)	85 (6.8)	3 (0.8)	28 (1.7)	3 (0.5)	3 (0.4)	0 (0.0)	26 (4.0)	28 (4.7)
≥2 RF (%)	29.3	7.5	7.2	4.3	5.1	1.4	17.0	17.3
Total physical activity (cpm)	456 (185)	684 (202)	493 (240)	623 (250)	532 (242)	632 (216)	460 (194)	428 (194)
MVPA (min/d)	47 (26)	63 (28)	44 (30)	62 (36)	49 (32)	70 (31)	39 (28)	33 (24)
VPA (min/d)	17 (15)	18 (13)	13 (13)	19 (17)	13 (13)	21 (15)	12 (13)	9 (10)
Sedentary (min/d)	466 (82)	318 (69)	419 (122)	347 (106)	390 (120)	425 (95)	419 (95)	421 (101)

Data are presented as mean (standard deviation) or median (25th‐75th percentile) unless otherwise stated. BMI, body mass index; cpm, counts per minute; Cross, cross‐sectional; DBP, diastolic blood pressure; RF, risk factor; Inter, interventional; Long, longitudinal; MetS, metabolic syndrome; MVPA, moderate‐ to vigorous‐intensity physical activity; SBP, systolic blood pressure; TG, triglycerides; VPA, vigorous‐intensity physical activity; WC, waist circumference.

One hundred and seventy‐six children and adolescents had MetS (2.9%). 2156 (35.9%) individuals had at least one, 601 (10.0%) at least two, 155 (2.6 %) at least three, 28 (0.5%) at least four features and four individuals (0.1%) all five features of MetS. The most prominent feature was abdominal obesity (34.2%), followed by low HDL‐cholesterol (10.2%), elevated fasting plasma glucose (8.9%), elevated blood pressure (8.7%) and elevated triglycerides (3.8%). MetS (3.8% vs. 2.1% p < 0.001), elevated blood pressure (12.1% vs. 5.6%, p < 0.001), fasting plasma glucose (11.9% vs. 6.1%, p < 0.001) and low HDL‐cholesterol (13.3% vs. 7.4%, p < 0.001) were more prevalent in boys, central obesity was more prevalent in girls (25.2% vs. 42.6%, p < 0.001) and elevated triglycerides (3.5% vs. 4.1%, p = 0.27) were equally prevalent in boys and girls (Table S2). The highest prevalence of MetS was seen in the UK (ALSPAC: 6.8%) and no cases were identified in Switzerland (KISS). To rule out the influence of age and because UK individuals were all between 15 years to 16 years old we compared the prevalence of MetS and its features when possible with results from the other studies in this age group (Table S3). Adolescents from the UK and the U.S. showed the highest prevalence of MetS (ALSPAC: 6.8%, NHANES 03/04: 5.2% and NHANES 05/06: 4.0%) and high rates of central obesity in this subgroup (ALSPAC: 50.1%, NHANES 03/04: 47.6% and NHANES 05/06: 46.3%).

Accelerometers were worn for an average of 4.8 days with a median wear time of 795 min/d (740 and 842 min/d for the 25^th^ and 75^th^ percentile). 91.7% of children and adolescents provided 3 or more valid days of accelerometer measurement. Total activity in boys was significantly higher than in girls and boys spent approximately 50% more time in MVPA and twice as much in VPA per day. Additionally, boys spent 6% less time per day sedentary (Table S2).

**Figure 1:**
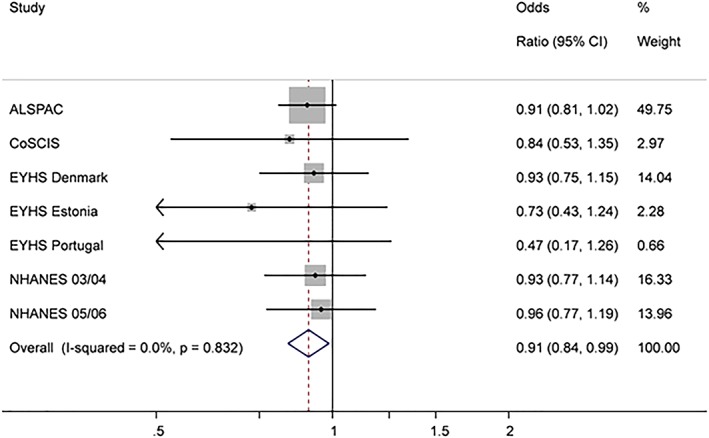
Associations between MVPA and MetS (after adjustment for sedentary time). Odds ratio represents a 10 min increase in MVPA. The model is adjusted for age, sex, monitor wear time and sedentary time

Results of the random‐effects meta‐analysis are shown in Table 2. Total physical activity was inversely associated with MetS (Fig. S1). For every 100 cpm increase in total physical activity the odds of MetS decreased by 17% [OR 0.83, 95% CI 0.76‐0.91]. MVPA and VPA were both inversely associated with MetS. A 10 min increase in MVPA resulted in 12 % [OR 0.88, 95% CI 0.82‐0.94] and a 10 min increase in VPA in 20% [OR 0.80, 95% CI 0.70‐0.92] reduced odds of MetS. After additional adjustment for sedentary time the association between MVPA and MetS was slightly attenuated but remained statistically significant (Fig. 1) [OR 0.91, 95% CI 0.84‐0.99] whereas the association between VPA and MetS became statistically insignificant (Fig. S2) [OR 0.86, 95% CI 0.71‐1.04].

**Table 2:** Associations between total physical activity, MVPA, VPA and sedentary time with MetS in 6009 children

	Odds ratio (95% CI)	Mutually adjusted OR (95% CI)
Total physical activity	0.83 (0.76‐0.91)	
MVPA	0.88 (0.82‐0.94)	0.91 (0.84‐0.99)
VPA	0.80 (0.70‐0.92)	0.86 (0.71‐1.04)
SED	1.28 (1.13‐1.45)	1.14 (0.96‐1.36)* 1.17 (0.97‐1.41)†

Total PA model was adjusted for sex and age; all other models were adjusted for sex, age, and monitor wear time. ORs represent a 100cpm increase in Total PA, 10 min increase in MVPA and VPA and 60 min increase in SED. MVPA, moderate‐ to vigorous‐intensity physical activity; SED, sedentary time; VPA, vigorous‐intensity physical activity.

* Additionally adjusted for MVPA.

†Additionally adjusted for VPA.

**Figure 2:**
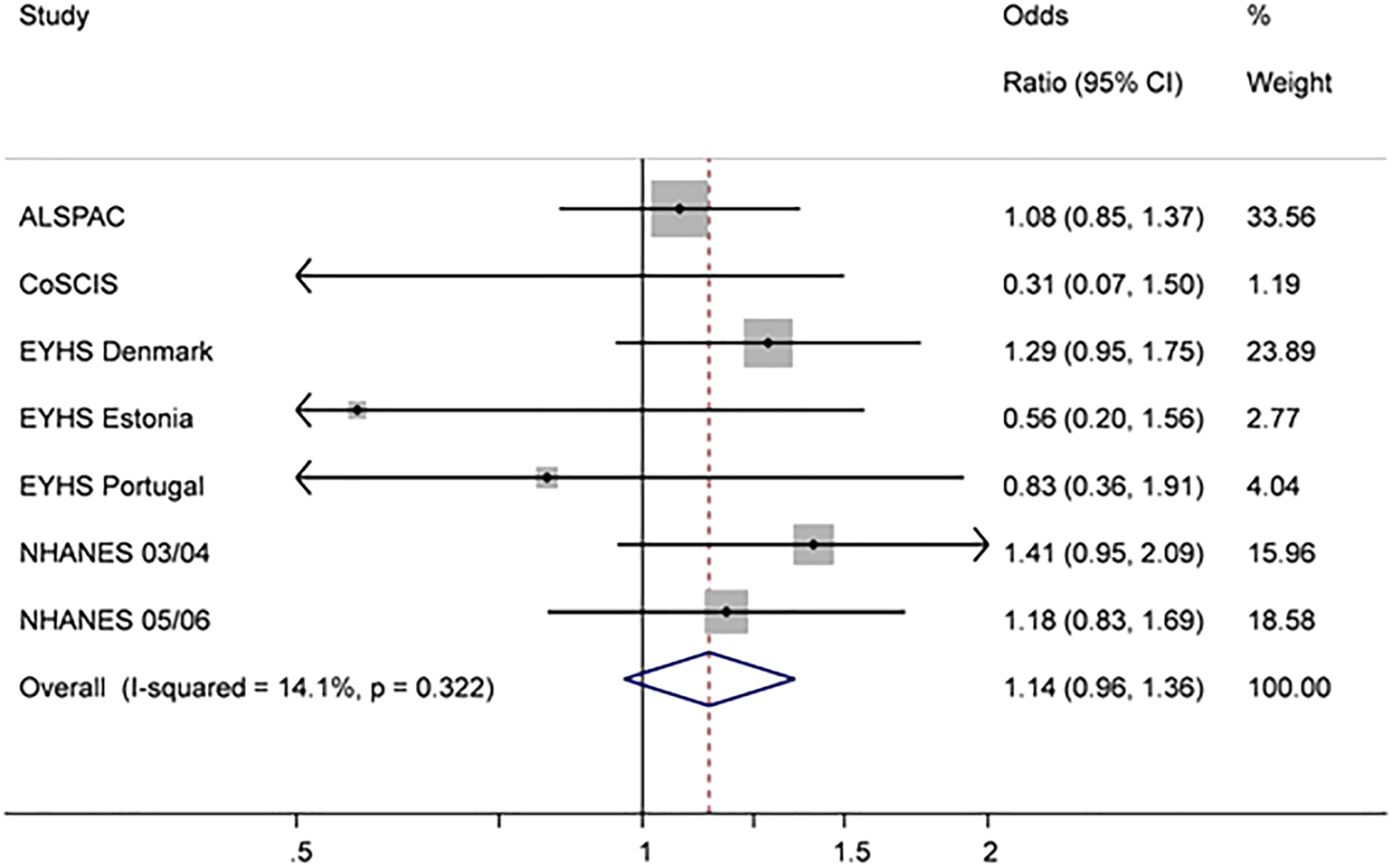
Association between sedentary time and MetS (after adjustment for MVPA). Odds ratio represents a 60 min increase in sedentary time. The model is adjusted for age, sex, monitor wear time and MVPA

Sedentary time was positively associated with MetS in the basic model. One hour of increased daily sedentary time resulted in 28% higher odds of MetS [OR 1.28, 95% CI 1.13‐1.45]. After additional adjustment for MVPA and VPA respectively the association between sedentary time and MetS was attenuated and not statistically significant (Fig. 2) [OR 1.14 95% CI 0.96‐1.36 and OR 1.17, 95% CI 0.97‐1.41].

Sensitivity analyses including individuals with at least 2, 3 and 4 valid days of accelerometer data showed virtually unchanged results.

## 4 DISCUSSION

The overall prevalence of MetS was low but differed notably between studies. Total physical activity and time spent in MVPA were inversely associated with MetS and the relationship between MVPA and MetS was independent of sedentary time. Sedentary time was not associated with MetS after adjustment for MVPA or VPA, respectively.

The prevalence of MetS we found (2.9%) was comparable to studies using the same definition of MetS in Turkish (2.3%), Vietnamese (4.6%), Malaysian (2.6%) and U.S. American children and adolescents (4.5%).^3^, ^22^, ^38^, ^39^ However, the prevalence differed considerably between the studies included in our sample. While it was low in Estonian, Danish, Portuguese and Swiss individuals, the prevalence in British and U.S. children and adolescents was comparably higher (Table 1). Adolescents in the ALSPAC study had relatively high blood pressure values. Around 30% of the individuals were categorized as hypertensive according to IDF criteria. However, methods of blood pressure measurement did not differ from other studies and individuals showed comparably high blood pressure in a subsequent wave of ALSPAC (data not shown).

The associations between objectively measured physical activity, sedentary time and MetS extend previous findings. MVPA was associated with MetS in a Vietnamese sample where adolescents in the least active quintile were 5‐times as likely to have MetS as adolescents in the most active quintile. However, adjustment for sedentary time was missing.^22^ In a sample of European youth, a 100 CPM higher total physical activity was associated with 30% lower odds of having MetS as compared to 17% in the present study. The differences might be explained by a different and more diverse sample in the present study and by different non‐wear time criteria.^1^ MVPA was associated with MetS independent of sedentary time in a sample of Latino and African–American youth.^23^ However, self‐reported sedentary time, operationalised as TV viewing and time spent on the phone, but not by accelerometer, was associated with MetS even after adjustment for MVPA.^23^ Several previous studies have suggested an association between screen time, especially TV viewing, and metabolic risk factors, whereas total sedentary time measured by accelerometers appears not associated with metabolic risk.^11^, ^16^, ^40^ TV viewing time constitutes only a part of the daily time spent sedentary and is associated with poor dietary habits which might partly explain the increased risk.^41^ VPA showed high variability in our sample which resulted in wider confidence intervals than MVPA when it was modelled as exposure variable and might explain the non‐significant finding for an association with MetS after adjustment for sedentary time

Atkin et al.^42^ examined the use of different cut‐points and non‐wear time criteria on the association between sedentary time and clustered metabolic risk in the European Youth Heart Study. After adjustment for total physical activity associations in most models were attenuated although statistically significant. However, when using the same non‐wear criteria and cut‐off points as in the present study, sedentary time was not associated with clustered metabolic risk after adjustment for total physical activity. In those models where sedentary time remained statistically significant with clustered metabolic risk even after adjustment for physical activity, either shorter periods of zero‐counts counted as non‐wear time (10min and 20min instead of 60min) or higher cut‐points (500cpm, 800cpm or 1000cpm instead of 100cpm) for sedentary time were used.^42^ Although the most optimal threshold used to define sedentary time from accelerometry is debated, it is likely higher cut‐points than 100 CPM increase the risk for misclassification.^43^ Further, non‐wear criteria of at least 60 minutes of consecutive zero‐counts are recommended.^44^

Several studies have suggested associations between objectively measured sedentary time and cardiometabolic risk factors, but these associations seem to disappear when controlling for MVPA.^18^, ^19^, ^42^ Similarly, our data suggests that the association between sedentary time and MetS is attenuated and non‐significant following adjustment for time spent in MVPA. Thus, sedentary time appears not an independent risk factor for MetS in children and adolescents.

We were unable to include data from the KISS study in our meta‐analysis since there were no cases of MetS in this study. Individuals in this study spent more time in MVPA and VPA than individuals in the other studies included, suggesting a healthier sample overall. Furthermore, ALSPAC had a comparably high weighing in the meta‐analysis (weights were estimated according to the inverse variance). However, the I^2^ value was low in all models, indicating low heterogeneity between studies.

The excluded children and adolescents were younger and more active (Table S1). This may have led to a slight overestimation of the prevalence of MetS overall, as MetS is associated with older age and physical inactivity.^1^ As exclusion of children and adolescents solely depended on data completeness and was not triggered by MetS status we do not assume that this systematically biased the found relationship between physical activity, sedentary time and MetS.

Strengths of this study include the diverse and large sample. That allowed us to treat MetS as a dichotomous outcome even although the prevalence of MetS was low. The heterogeneity of the sample is another advantage and increases, in combination with our meta‐analytical approach, external validity. Sedentary time and physical activity were measured objectively resulting in more precise estimates for their association with MetS compared to physical activity estimates derived from questionnaires.

Potential limitations of this study should be taken into account when interpreting the results. Due to the cross‐sectional design, causality cannot be inferred. There is however some evidence suggesting a prospective relationship between physical activity and several features of MetS in children and adolescents,^20^, ^45^ while sedentary time shows no such association.^11^ Although physical activity and sedentary time were measured objectively, there are some limitations of this method. Due to its mode of operation, accelerometers cannot distinguish between time spent sitting and time spent standing which might lead to some misclassification. Additionally, activities like cycling or swimming are recorded inadequately. Accelerometer measurements involve some subjective decisions that can influence the association between exposure and outcome. However, as previously discussed, the cut‐off points and non‐wear‐time criteria we used seem realistic,^32^ and our decision to include all individuals with at least one day of at least 8h monitor wear time seems robust. Sensitivity analyses in individuals providing at least 2, 3 or 4 valid days did not change our results notably (Table S4). Finally, although less likely, we cannot exclude the possibility that our results are explained by unmeasured confounding factors such as dietary intake, socio‐economic status and genotype.

The prevalence of MetS overall was low, but differed substantially between the single studies. MVPA was associated with MetS in children and adolescents independent of sedentary time. Sedentary time was not associated with MetS after adjustment for MVPA or VPA. These findings suggest that the promotion of physical activity might be more useful as a public health focus than the reduction of sedentary time.

## CONFLICT OF INTEREST

No conflict of interest was declared.

## FUNDING

The pooling of the data was funded through a grant from the National Prevention Research Initiative (Grant Number: G0701877) (http://www.mrc.ac.uk/research/initiatives/national-prevention-research-initiative-npri/). The funding partners relevant to this award are British Heart Foundation, Cancer Research UK, Department of Health, Diabetes UK, Economic and Social Research Council, Medical Research Council, Research and Development Office for the Northern Ireland Health and Social Services, Chief Scientist Office, Scottish Executive Health Department, The Stroke Association, Welsh Assembly Government and World Cancer Research Fund. This work was additionally supported by the Medical Research Council (MC_UU_12015/3; MC_UU_12015/7), The Research Council of Norway (249932/F20), Bristol University, Loughborough University and Norwegian School of Sport Sciences.

## The ICAD Collaborators include the following

Prof LB Andersen, Department of Teacher Education and Sport, Western Norwegian University of Applied Sciences, Sogndal, Norway [Copenhagen School Child Intervention Study (CoSCIS)]; Prof S Anderssen, Norwegian School for Sport Science, Oslo, Norway (European Youth Heart Study (EYHS), Norway); Dr AJ Atkin, Faculty of Medicine and Heath Sciences, University of East Anglia, UK; Prof G Cardon, Department of Movement and Sports Sciences, Ghent University, Belgium (Belgium Pre‐School Study); Centers for Disease Control and Prevention (CDC), National Center for Health Statistics (NCHS), Hyattsville, MD USA [National Health and Nutrition Examination Survey (NHANES)]; Dr R Davey, Centre for Research and Action in Public Health, University of Canberra, Australia [Children's Health and Activity Monitoring for Schools (CHAMPS)]; Prof U Ekelund, Norwegian School of Sport Sciences, Oslo, Norway; Dr DW Esliger, School of Sports, Exercise and Health Sciences, Loughborough University, UK; Dr P Hallal, Postgraduate Program in Epidemiology, Federal University of Pelotas, Brazil (1993 Pelotas Birth Cohort); Dr BH Hansen, Norwegian School of Sport Sciences, Oslo, Norway; Prof KF Janz, Department of Health and Human Physiology, Department of Epidemiology, University of Iowa, Iowa City, US (Iowa Bone Development Study); Prof S Kriemler, Epidemiology, Biostatistics and Prevention Institute, University of Zürich, Switzerland [Kinder‐Sportstudie (KISS)]; Dr N Møller, University of Southern Denmark, Odense, Denmark (European Youth Heart Study (EYHS), Denmark); Dr K Northstone, School of Social and Community Medicine, University of Bristol, UK [Avon Longitudinal Study of Parents and Children (ALSPAC)]; Dr A Page, Centre for Exercise, Nutrition and Health Sciences, University of Bristol, UK [Personal and Environmental Associations with Children's Health (PEACH)]; Prof R Pate, Department of Exercise Science, University of South Carolina, Columbia, US [Physical Activity in Pre‐school Children (CHAMPS‐US) and Project Trial of Activity for Adolescent Girls (Project TAAG)]; Dr JJ Puder, Service of Endocrinology, Diabetes and Metabolism, Centre Hospitalier Universitaire Vaudois, University of Lausanne, Switzerland (Ballabeina Study); Prof J Reilly, Physical Activity for Health Group, School of Psychological Sciences and Health, University of Strathclyde, Glasgow, UK [Movement and Activity Glasgow Intervention in Children (MAGIC)]; Prof J Salmon, Institute for Physical Activity and Nutrition (IPAN), School of Exercise and Nutrition Sciences, Deakin University, Geelong, Australia [Children Living in Active Neigbourhoods (CLAN) & Healthy Eating and Play Study (HEAPS)]; Prof LB Sardinha, Exercise and Health Laboratory, Faculty of Human Movement, Universidade de Lisboa, Lisbon, Portugal (European Youth Heart Study (EYHS), Portugal); Dr LB Sherar, School of Sports, Exercise and Health Sciences, Loughborough University, UK; Dr EMF van Sluijs, MRC Epidemiology Unit & Centre for Diet and Activity Research, University of Cambridge, UK [Sport, Physical activity and Eating behaviour: Environmental Determinants in Young people (SPEEDY)].

## Supporting information


**Table S1.** Comparison of included and excluded individuals
**Table S2.** Descriptive Characteristics of Participants Stratified by Sex
**Table S3.** Prevalence of MetS and features of MetS in 15‐16y olds
**Table S4.** Associations between Total Physical Activity, MVPA, VPA, and Sedentary Time With MetS where only children with at least 2, 3 or 4 valid days of accelerometer measurement were included
**Figure S1**. Forest plot for the associations between total physical activity (Counts per minute) and the metabolic syndrome. Data are Odds Ratios (95% CI)
**Figure S2**. Forest plot for the associations between vigorous intensity physical activity adjusted for sedentary time and the metabolic syndrome. Data are Odds Ratios (95% CI).
