# Supplementary material for: Associations between accelerometry measured physical activity and sedentary time and the metabolic syndrome: A meta‐analysis of more than 6000 children and adolescents
Source: Pediatr Obes. 2019 Nov 10;15(1):e12578. doi: 10.1111/ijpo.12578 (PMC7003500; doi:10.1111/ijpo.12578)

SUPPLEMENTARY MATERIAL

Associations between accelerometry measured physical activity and sedentary time and the metabolic syndrome: A meta-analysis of more than 6000 children and adolescents

Marius Renninger[1,2], Bjørge H. Hansen[1], Jostein Steene-Johannessen[1], Susi Kriemler[3], Niels Christian Møller[4], Kate Northstone[5], Luis Sardinha[6], Sigmund Anderssen[1], Lars Bo Andersen[7], Ulf Ekelund[1,4], On behalf of the International Children's Accelerometry Database (ICAD) Collaborators

[1] Department of Sport Medicine, Norwegian School of Sport Sciences, Oslo, Norway

[2] Department of Medical Informatics, Biometry and Epidemiology (IBE), Ludwig-Maximilians-Universität München, Munich, Germany

[3] Epidemiology, Biostatistics and Public Health Institute, University of Zürich, Switzerland

[4] University of Southern Denmark, Odense, Denmark

[5] School of Social and Community Medicine, University of Bristol, UK

Exercise and Health Laboratory, CIPER, Fac Motricidade Humana, Universidade de Lisboa

[7] Faculty of Education, Arts and Sport, Western Norway University of Applied Sciences, Sogndal, Norway

[8] Norwegian Institute for Public Health, Oslo, Norway

Supplementary Table S 1. Comparison of included and excluded individuals

	Excluded	Included	p-value
Age	13.5 (1.9)	14.0 (2.6)	<0.001
% female	51 (0.5)	51 (0.5)	
DBP	60.4 (9.1)	61.6 (8.4)	<0.001
Glucose	5.2 (0.7)	5.1 (0.5)	<0.001
HDL	1.4 (0.3)	1.4 (0.3)	<0.001
Insulin	72.2 (45.1)	64.1 (45.6)	<0.001
LDL	2.3 (0.6)	2.3 (0.6)	0.50
SBP	109.8 (14.4)	110.2 (12.6)	0.11
Triglycerides	1.1 (1.0)	0.8 (0.4)	<0.001
Waist circumference	72.2 (11.3)	71.2 (12.2)	<0.001
BMI	20.6 (4.2)	20.6 (4.3)	0.67
Total PA	533.1 (212.4)	501 (226.0)	<0.001
MVPA	50.2 (28.7)	46.7 (30.4)	<0.001
VPA	15.8 (13.7)	14.6 (14.1)	<0.001
Sedentary	393.5 (95.4)	418.2 (110.3)	<0.001

Data are presented as mean (standard deviation) unless otherwise stated; abbreviations: DBP, diastolic blood pressure; HDL, high-density lipoprotein; LDL, low-density lipoprotein, BMI, body mass index; PA, physical activity; MVPA, moderate- to vigorous-intensity physical activity; VPA, vigorous-intensity physical activity;

Supplementary Table S 2. Descriptive Characteristics of Participants Stratified by Sex

	Boys (n=2876)	Girls (n= 3133)	p-value
Age (years) median (IQR)	15.3 (10.2-15.7)	15.3 (11.0-15.7)	0.17
Weight (kg)	56.1 (18.8)	52.6 (15.2)	< 0.001
Height (cm)	163.4 (16.7)	157.6 (12.3)	< 0.001
Waist circumference(cm)	71.9 (12.4)	70.6 (12.1)	< 0.001
BMI (kg/m^2)	20.4 (4.3)	20.8 (4.4)	< 0.001
Birthweight (g)	3481 (601)	3367 (532)	< 0.001
SBP (mm Hg)	112.3 (13.1)	108.1 (11.7)	< 0.001
DBP (mm Hg)	60.9 (9.2)	62.1 (7.8)	< 0.001
HDL Cholesterol (mmol/l)	1.4 (0.3)	1.4 (0.3)	< 0.001
Glucose (mmol/l)	5.2 (0.5)	5.0 (0.5)	< 0.001
Insulin (pmol/l) median (IQR)	49.8 (34.0 – 70.9)	59.3 (42.0 – 83.0)	< 0.001
Triglycerides (mmol/l) median (IQR)	0.70 (0.53 – 0.94)	0.76 (0.58-1.0)	< 0.001
Total activity (cpm)	561 (241)	445 (195)	< 0.001
Sedentary Time (min/d)	403 (112)	431 (107)	< 0.001
MVPA (min/d)	56 (33)	38 (25)	< 0.001
VPA (min/d)	18 (16)	10 (11)	< 0.001

Data are presented as mean (standard deviation) unless otherwise stated; abbreviations: BMI, body mass index; SBP, systolic blood pressure; DBP, diastolic blood pressure; HDL, high-density lipoprotein; IQR, interquartile range;

Supplementary Table S 3. Prevalence of MetS and features of MetS in 15-16y olds

Study	N	boys	MetS	WC	HDL	BP	TG	BG
ALSPAC	1251	44.6	6.8 (5.5-8.3)	50.1 (47.4-52.9)	17.9 (15.9-20.1)	30.1 (27.6-32.7)	2.9 (2.1-4.0)	10.6 (9.0-12.5)
EYHS Denmark	982	45.3	2.4 (1.6-3.6)	24.0 (21.5-26.8)	12.4 (10.5-14.6)	6.2 (4.9-7.9)	5.3 (4.1-6.9)	8.5 (6.9-10.4)
EYHS Estonia	232	41.4	0.9 (0.2-3.3)	8.6 (5.7-12.9)	10.3 (7.1-14.9)	6.9 (4.3-10.9)	1.7 (0.7-4.3)	6.0 (3.6-9.9)
EYHS Portugal	305	48.9	0.7 (0.2-2.4)	10.8 (7.8-14.8)	9.8 (7.0-13.7)	2.0 (0.9-4.2)	1.3 (0.5-3.3)	12.1 (8.9-16.3)
NHANES 03/04	191	61.8	5.2 (2.9-9.4)	47.6 (40.7-54.7)	11.5 (7.7-16.8)	4.7 (2.5-8.7)	10.5 (6.9-15.6)	9.4 (6.0-14.4)
NHANES 05/06	177	48.0	4.0 (1.9-7.9)	46.3 (39.1-53.7)	8.5 (5.2-13.5)	4.5 (2.3-8.7)	5.6 (3.1-10.1)	13.0 (8.8-18.7)

Data are presented in percentages (95% CI) unless otherwise stated; abbreviations: MetS, metabolic syndrome; WC, waist circumference; HDL, high-density lipoprotein; BP, blood pressure; TG, triglycerides; BG, blood glucose

Supplementary Table S 4. Associations between Total Physical Activity, MVPA, VPA, and Sedentary Time With MetS where only children with at least 2, 3 or 4 valid days of accelerometer measurement were included

**At least 2 valid days**	**Odds Ratio (95% CI)**	**Mutually adjusted OR (95% CI)**
Total Physical Activity	0.82 (0.74-0.90)	
MVPA	0.87 (0.81-0.94)	0.91 (0.84-0.99)
VPA	0.79 (0.69-0.91)	0.85 (0.70-1.04)
SED	1.30 (1.14-1.47)	1.14 (0.96-1.36)*
		1.19 (0.98-1.44)†
**At least 3 valid days**		
Total Physical Activity	0.80 (0.73-0.89)	
MVPA	0.86 (0.80-0.93)	0.89 (0.82-0.97)
VPA	0.79 (0.68-0.91)	0.85 (0.72-1.01)
SED	1.30 (1.14-1.48)	1.14 (0.97-1.34)*
		1.19 (0.99-1.43)†
**At least 4 valid days**		
Total Physical Activity	0.80 (0.72-0.89)	
MVPA	0.86 (0.80-0.93)	0.91 (0.84-0.99)
VPA	0.80 (0.68-0.93)	0.86 (0.71-1.04)
SED	1.32 (1.15-1.52)	1.18 (1.00-1.39)*
		1.22 (1.02-1.46)†

Total PA model was adjusted for sex & age, all other models were adjusted for sex, age, monitor wear time. Abbreviations: MVPA, moderate- to vigorous-intensity physical activity; VPA, vigorous-intensity physical activity; SED, sedentary time. ORs represent a 100cpm increase in Total PA, 10 min increase in MVPA&VPA and 60min increase in SED. *additionally adjusted for MVPA †additionally adjusted for VPA

Supplementary Figure 1. Forest plot for the associations between total physical activity (Counts per minute) and the metabolic syndrome. Data are Odds Ratios (95% CI)

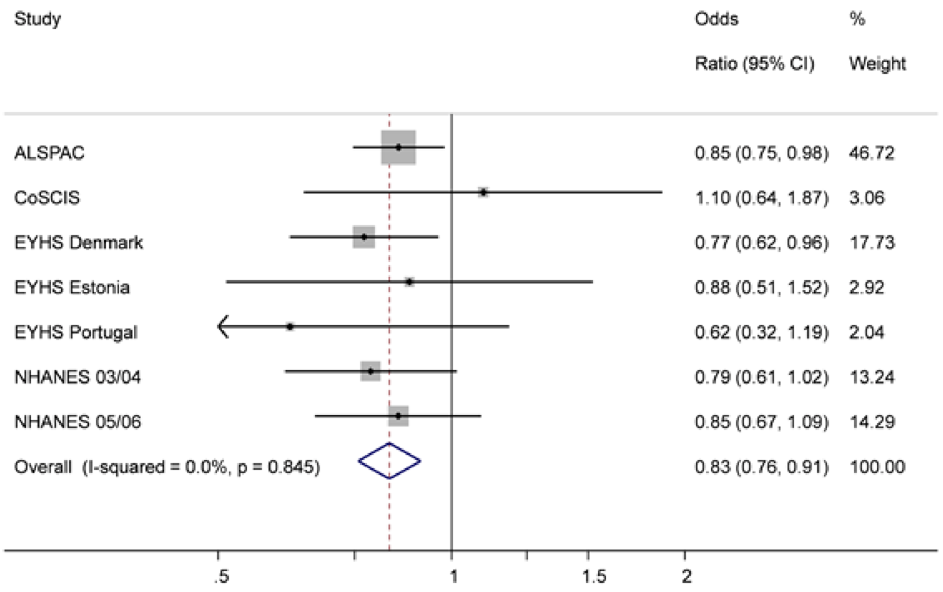

Supplementary Figure 2. Forest plot for the associations between vigorous intensity physical activity adjusted for sedentary time and the metabolic syndrome. Data are Odds Ratios (95% CI).

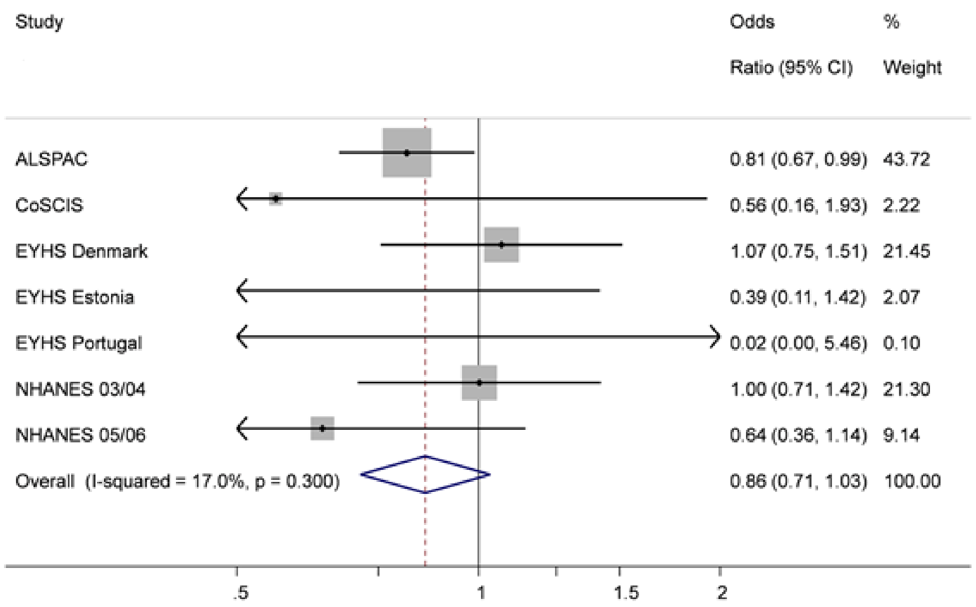

Supplement: Supplementary file 1 — Table S1. Comparison of included and excluded individuals Table S2. Descriptive Characteristics of Participants Stratified by Sex Table S3. Prevalence of MetS and features of MetS in 15‐16y olds Table S4. Associations between Total Physical Activity, MVPA, VPA, and Sedentary Time With MetS where only children with at least 2, 3 or 4 valid days of accelerometer measurement were included Figure S1. Forest plot for the associations between total physical activity (Counts per minute) and the metabolic syndrome. Data are Odds Ratios (95% CI) Figure S2. Forest plot for the associations between vigorous intensity physical activity adjusted for sedentary time and the metabolic syndrome. Data are Odds Ratios (95% CI). [file IJPO-15-e12578-s001.pdf]
